# Comparison of Optical and Stylus Methods for Surface Texture Characterisation in Industrial Quality Assurance of Post-Processed Laser Metal Additive Ti-6Al-4V

**DOI:** 10.3390/ma16134815

**Published:** 2023-07-04

**Authors:** Theresa Buchenau, Tobias Mertens, Hubertus Lohner, Hauke Bruening, Marc Amkreutz

**Affiliations:** 1 Fraunhofer Institute for Manufacturing Technology and Advanced Materials IFAM, 28359 Bremen, Germany; hauke.bruening@ifam.fraunhofer.de (H.B.); marc.amkreutz@ifam.fraunhofer.de (M.A.); 2 Airbus Operations, 28199 Bremen, Germany; tobias.mertens@airbus.com (T.M.); hubertus.lohner@airbus.com (H.L.)

**Keywords:** metal additive manufacturing, laser powder bed fusion, optical surface texture measurement, electro-chemical surface treatment, Ti6Al4V

## Abstract

Additive manufacturing technologies enable lightweight, functionally integrated designs and development of biomimetic structures. They contribute to the reduction of material waste and decrease in overall process duration. A major challenge for the qualification for aerospace applications is the surface quality. Considering Ti-64 laser powder bed fusion (LPBF) parts, particle agglomerations and resulting re-entrant features are characteristic of the upper surface layer. Wet-chemical post-processing of the components ensures reproducible surface quality for improved fatigue behaviour and application of functional coatings. The 3D SurFin^®^ and chemical milling treatments result in smoother surface finishes with characteristic properties. In order to characterise these surfaces, three methods for surface texture measurement (contact and non-contact) were applied, namely confocal microscopy, fringe projection and stylus profilometry. The aim of this work was to show their suitability for measurement of laser powder bed fusion as-built and post-processed surfaces and compare results across the evaluated surface conditions. A user-oriented rating of the methods, summarising advantages and disadvantages of the used instruments specifically and the methods in general, is provided. Confocal microscopy reaches the highest resolution amongst the methods, but measurements take a long time. The raw data exhibit large measurement artefacts for as-built and chemically milled conditions, requiring proper data post-processing. The stylus method can only capture 2D profiles and the measurement was restricted by particle agglomerations and craters. However, the method (process and instrument) is entirely standardised and handheld devices are inexpensive, making it accessible for a large group of users. The fringe projection method was the quickest and easiest regarding measurement and data post-processing. Due to large areal coverage, reproduction of location when performing repeat measurements is possible. The spatial resolution is lower than for confocal microscopy but is still considered sufficiently high to characterise the investigated surface conditions.

## 1. Introduction

The maturation of metal additive manufacturing (AM) technologies offers new opportunities for the aerospace industry. The dimensional freedom enables lightweight designs and development of biomimetic structures, contributes to the reduction of material waste and decrease in manufacturing process duration [1,2,3].

The titanium–aluminium–vanadium alloy Ti-6Al-4V (Ti-64) is a popular aerospace alloy and has various load bearing applications in airplane and satellite structures [4,5,6,7]. Therefore, it is also a common research material in metal additive manufacturing [2,8,9,10,11,12,13].

One of the most common metal AM processes is laser powder bed fusion. Typical features that may be observed on as-built surfaces include powder particle agglomerations, re-entrant features or weld tracks. The near surface region may exhibit open and closed pores. This characteristic quality imposes new challenges on surface topography measurements, part qualification and quality assurance (QA) processes.

Metal AM parts are particularly interesting for load bearing applications, where surface finish might be critical for part qualification, and manufacturing of complex geometries. Therefore, wet-chemical surface treatment methods, such as electropolishing and chemical milling, seem appropriate [8,14,15]. These methods also accommodate material removal to eliminate near surface defects.

In order to properly characterise as-built and post-treated parts, different methods for surface topography measurement can be applied. The most widely known and applied method is contact stylus profilometry, which captures 2D linear height profiles. In recent years, optical measurement systems are increasingly gaining acceptance and are attractive regarding areal characterisation to obtain a statistically more representative depiction of the surfaces considered [16,17,18]. In this work, the comparability of results from selected optical methods to those from the well-known stylus method is investigated and the applicability of the chosen measurement methods to as-built and post-treated AM surfaces is tested.

Previous studies found in the literature on comparison of measurement methods for application to AM parts focussed on multiple non-contact areal methods [19,20,21,22].

Thompson et al. and Senin et al. compared coherence scanning interferometry (CSI), confocal microscopy (CM), focus variation (FV) and X-ray computed tomography (xCT). They looked into surface topography using data sets from the different measurement techniques for one as-built Ti-64 sample. That way they studied aligned profiles extracted from areal measurements, areal texture parameters and reconstruction of typical metal powder bed fusion areal features such as powder particle agglomerations, weld tracks and others, by those methods [19,20]. De Pastre et al. applied the same measurement methods to characterise an as-built polymer powder bed fusion sample and additionally included stylus profiles. They found that computed profile texture parameters from stylus were lower than from non-contact methods [22].

Tato et al. used a combined CSI, CM and FV instrument and compared evaluated areal parameters for co-located data sets on one vertical and one horizontal as-built 316L surface. They found that the CSI measurement was the most time consuming. The variations for most computed areal texture parameters were below 8% [21].

Whip et al. used fringe projection and (destructive) cross section analysis to characterise multiple as-built Inconel 718 surface conditions and found that texture parameters from fringe projection did not accurately represent maximum valley depth. They attributed this matter to residual loose powder and shadowing effects from powder particle agglomerations [23].

This work aims to assess the applicability and comparability of the chosen surface texture characterisation methods when applied for quality assurance (QA). It is explicitly not intended to compare the data of identical locations to show the difference in profile measured by the selected methods, but to address challenges during a realistic QA process.

In addition to the as-built surface condition, post-processed Ti-64 AM samples are examined, namely chemically milled (ChM), electropolished by 3D SurFin (3DS) and a combined treatment (3DS+).

In 2017, Todhunter et al. published a survey on the use of profile and areal surface texture parameters in research and industry. They found that Ra (arithmetic mean height) and Rt (total maximum height) were still among the most frequently used parameters, especially in automotive, aerospace and product manufacturing [18]. The parameters are also applied in recent publications on metal AM surface texture, sometimes in combination with their areal equivalents, and are generally well known in research and industry [17,24,25,26,27,28].

Therefore, the arithmetic mean height Ra and maximum total height Rt are adopted in this paper to make the results accessible and comprehensible for a broad range of readers. Another reason for choosing profile characterisation was to be able to include the widely familiar stylus profilometry method in the comparison.

## 2. Materials and Methods

**Figure 1:**
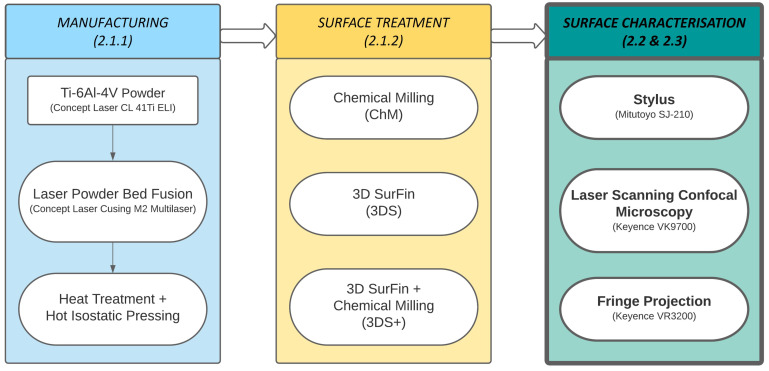
Overview: manufacturing, surface treatment and surface characterisation of the evaluated samples. Surface characterisation (left box) is this work’s focus; numbers indicate the corresponding sections.

This section includes a description of the evaluated samples with manufacturing and surface treatment steps (see Figure 1), information on the utilised surface topography measurement systems, measurement settings, data processing and evaluation steps.

### 2.1. Samples

**Figure 2:**
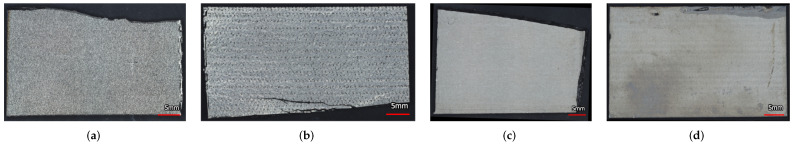
Sample photographs: (**a**) AsB—As-built surface condition; (**b**) ChM—after chemical milling treatment; (**c**) 3DS—after 3D SurFin^®^ treatment; (**d**) 3DS+—after combined 3D SurFin^®^ and chemical milling treatment.

**Figure 3:**
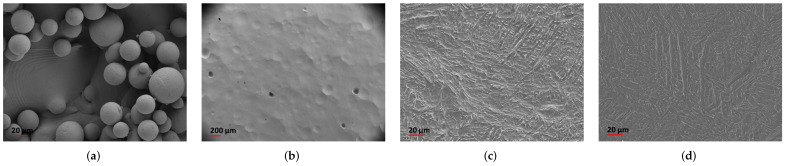
Images from scanning electron microscopy (SEM). (**a**) AsB—typical powder particle agglomerations; (**b**) ChM—characteristic craters from particle removal; (**c**) 3DS—etching marks after peak reduction; (**d**) 3DS+—etching marks, similar to 3DS.

Figure 1 summarises the steps of manufacturing, surface treatment and characterisation methods for the used samples, whilst the focus of this work is on surface texture characterisation. The characterisation methods are applied to four different surface conditions of Ti-64 samples from LPBF. Conditions under investigation are as-built (AsB), after chemical milling (ChM), after 3D SurFin^®^ (3DS) and after combined 3D SurFin^®^ and chemical milling (3DS+). Figure 2 and Figure 3 show sample photographs and microscopic images, respectively, and sample sizes and surface conditions are given in Table 1.

#### 2.1.1. Manufacturing

The evaluated samples originate from a study on combined wet-chemical surface post-processing and were manufactured in an LPBF process on a Concept Laser Cusing M2 Multilaser using identical manufacturing parameters and Ti-6Al-4V powder (Concept Laser CL 41Ti ELI). After additive manufacturing, all samples were heat-treated for stress relief and hot isostatically pressed for bulk quality improvement.

#### 2.1.2. Surface Treatment

Surface treatments applied to each of the evaluated samples are specified in Table 1. The chemical composition of the bath and other specifics for both processes are summarised in Table 2 [8]. Brief descriptions of 3D SurFin^®^ and chemical milling processes are given subsequently. For detailed treatment process information, refer to [8].

##### 3D SurFin^®^

The enhanced electropolishing process 3D SurFin^®^ is a process specifically designed to remove peaks from a surface. It uses an electrolyte based on deionised water combined with ammonium fluoride, methylglycinediacetic acid and sulfuric acid and is operated at 80 °C [8,29]. The water-based electrolyte enables the application of higher voltages (200–400 V), leading to a shorter process duration for the same material removal rate [30,31].

##### Chemical Milling

Acidic etching baths for Ti-64 commonly consist of a mixture of distilled water, hydrofluoric acid HF and nitric acid $HNO_{3}$ , which is the recommended standard according to ASTM B600 [32]. The actual material removal is caused by the hydrofluoric acid, reacting with the titanium oxide on the surface and forming titanium fluoride and hydrogen. The nitric acid $HNO_{3}$ acts as an oxidant and is responsible for bonding the atomic hydrogen [32,33].

#### 2.1.3. Macroscopic and Microscopic Visual Inspection

**Figure 4:**
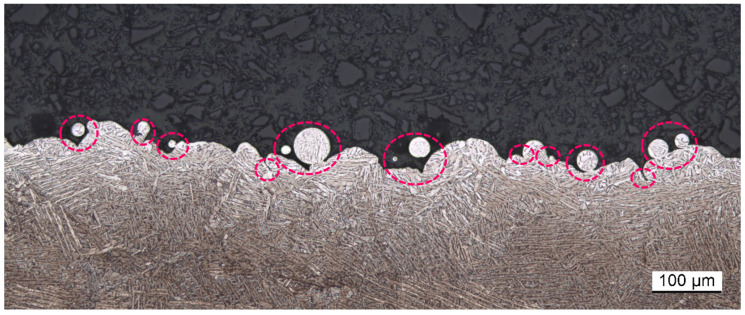
AsB—cross section micrograph; markers highlight re-entrant features which cannot be detected by contact and line-of-sight methods.

**Figure 5:**
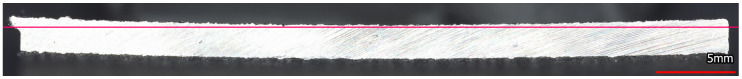
AsB—cross section curvature, caused by residual stresses.

From visual and microscopic inspection, the as-built (AsB) surface shows characteristic powder particle agglomerations that are typical for LPBF surfaces (Figure 2 and Figure 3), causing a high initial surface roughness [8,34]. Figure 3 and Figure 4 show these agglomerations of different sizes as well as resulting re-entrant features. The sample has a curvature (see Figure 5), which probably originates from residual stresses introduced during the manufacturing process.

**Figure 6:**
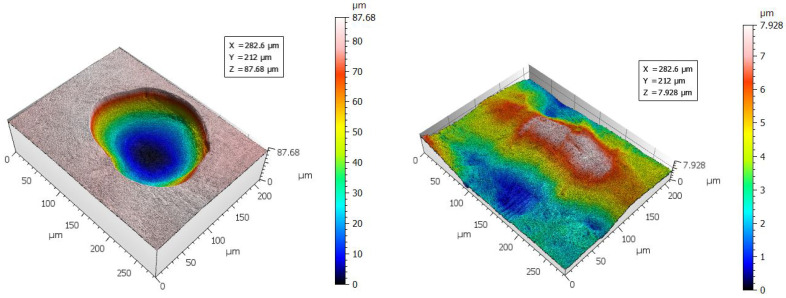
ChM-3D surface plots from LSCM: two measured positions show fundamentally different surface characteristics on the same sample.

After chemical milling (ChM) the surface exhibits craters and pits of various sizes and slopes (Figure 3). The linear pattern visible in the photo (Figure 2) corresponds to sequences of pits, similar to the one presented in Figure 6, supposedly originating from powder particles (as those present on the AsB surface) that were detached from the surface during the post-treatment process.

The 3DS sample was subjected to a 15 min 3D SurFin^®^ treatment. The 3DS+ sample received an additional 20 min chemical milling treatment, resulting in higher material removal. Therefore it is likely, that more subsurface flaws were removed [8,29]. With the bare eye, the 3DS and 3DS+ surfaces appear to be smooth (Figure 2); however, on a microscopic scale they show etching marks resulting from the different phases in the α−β− alloy Ti-64, appearing as random groove pattern (Figure 3).

### 2.2. Surface Texture Characterisation-Theory

The surface characterisation methods chosen for this study are stylus measurements, laser scanning confocal microscopy (LSCM) and fringe projection (FP). Physical working principles and previous applications to metal AM surfaces of the methods as well as selected surface texture parameters are described subsequently.

#### 2.2.1. Methods for Surface Texture Characterisation

##### Laser Scanning Confocal Microscopy (LSCM)

In LSCM (Keyence VK9700), a laser scans the surface in different focal planes while only exposing the area portion in focus. Vertical scanning of the entire surface depth and layering of the resulting images leads to a 3D surface representation. A lateral resolution in the range of several hundred nanometres can be achieved [35,36,37].

The LSCM can be used for areal surface characterisation according to ISO 25178 and is listed as one of the optical methods suitable for surface characterisation in that same standard [38,39]. Drawbacks of laser confocal microscopy are the long acquisition time in relation to the size of the measured area and the line-of-sight restriction, prohibiting the detection of re-entrant features (marked in Figure 4).

In the literature on AM surface characterisation, the method is mainly used for areal characterisation [9,20,37,40,41], but extracting and evaluating line profiles is possible.

##### Fringe Projection (FP)

**Figure 7:**
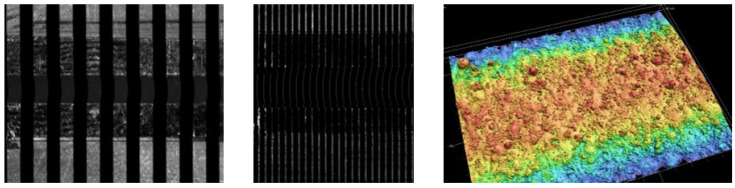
Fringe projection process—projected patterns (**left**,**middle**); screenshots during measurement) and resulting 3D surface plot (**right**).

The fringe projection method is based on the projection of fringe patterns of different sizes onto a surface as visualised in Figure 7. The deviation between the projected pattern and its appearance on the surface is measured and, by means of triangulation of object, camera and projector, the 3D surface is reconstructed [42,43]. The system (Keyence VR3200) measures 3D surface data of a representative surface portion within an acceptable period of time (i.e., 1 cm^2^ in five minutes or less, depending on the resolution) and can return standardised surface parameters according to ISO 25178 [38] from these data. In ISO 25178, fringe projection is listed as suitable method for areal surface texture measurement [36,39]. The extraction of line profile data and subsequent calculation of line roughness parameters according to ISO 21920-2 [44] is possible. The method is line-of-sight and therefore unable to detect re-entrant features (as those shown in Figure 4).

In the literature, the method is mostly used for geometrical shape measurements on different scales (down to the size of microfibres) [42,45,46,47] or in-process monitoring for LPBF [48,49,50,51]. Fringe projection was used for metal AM surface topography measurement by Whip et al. [23] and Zheng et al. in comparison with focus variation microscopy [43]. We previously applied the method for areal characterisation in relation with fatigue data [52,53].

##### Stylus Profilometry

The stylus method (Mitutoyo SJ-210 instrument) is a contact measurement method using a conical diamond tip that is moved across the surface, following its contour, leading to a 2D profile. Parameters, tip, data filtering and measurement procedure are standardised in ISO 21920-2, ISO 21920-3 and ISO 3274 [44,54,55].

**Figure 8:**
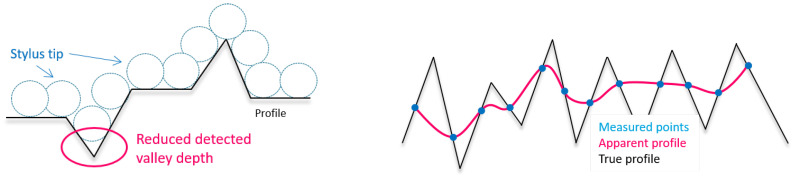
Reduced valley penetration depth due to stylus tip size (**left**); surface smoothing due to low data point density (**right**) based on [[56,66]].

The achievable resolution of the method is mainly dependent on the size of the tip and the density of data points, as illustrated in Figure 8. If the tip is too large, valleys cannot be penetrated and the surface appears to be smoother. Furthermore, the circular tip shape may result in rounded peaks, while peak height remains unaffected. If the data point density is too low, some peaks and valleys remain undetected, leading to data smoothing as well. Another contributing factor is the cone angle, which also has an influence on the penetration depth of narrow valleys. Moreover, the detection of re-entrant features is not possible [56,57].

Using too small a tip may result in its damage, as it will be subjected to a large force in horizontal direction when getting stuck in higher surface features [56]. ISO 3274, however, only specifies a minimum tip size required for a certain expected surface roughness [55].

Stylus measurements and the corresponding line parameters Ra and Rt are still industry standard and widely used across literature on surface characterisation in general [17,18]. The same is true for metal AM surface characterisation in particular. A few examples are cited subsequently [8,17,29,34,41,58,59,60,61,62,63,64,65].

#### 2.2.2. Parameters for Surface Texture Characterisation

The surface characterisation parameters selected for the method comparison are the arithmetic mean height of the roughness profile Ra and the maximum total height of the profile Rt . They were chosen because they are the established parameters for different industrial applications and are widely used throughout the literature to characterise metal AM surfaces [17,29,34,41,58].

### 2.3. Surface Texture Characterisation-Experimental Approach

In order to examine the applicability of the selected profile and areal surface characterisation methods, four samples of different surface condition have been selected for examination:

- Initial surface condition (AsB);
- Chemically milled surface condition (ChM);
- Surface condition after 3D SurFin^®^ (3DS);
- Surface condition after combined 3D SurFin^®^ and subsequent chemical milling (3DS+).

**Figure 9:**
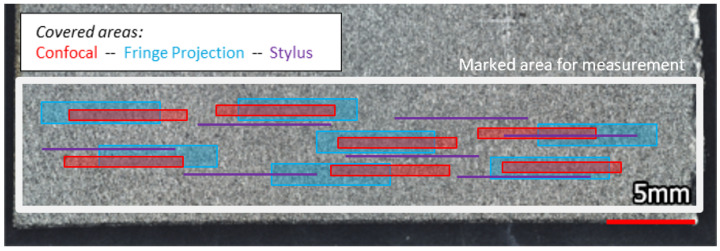
Data Acquisition—within a marked region on each sample, multiple measurements by every method were performed, distributed across the entire region.

On each sample, measurements were taken within a marked area. Due to instrument restrictions, area portions of varying size were covered. Specific values are indicated in Table 3. An illustration of different sizes of covered areas due to method-specific restrictions is given in Figure 9. Furthermore, the same low and high pass filters as specified in ISO 3274 [55] were applied to the data retrieved from all methods (see Table 4).

#### 2.3.1. Measurement Setup

The measured area length for the optical measurements of each sample was selected according to the expected surface roughness as specified in ISO 21920 [44,54] (includes, a.o., updates of the withdrawn profile surface texture standards ISO 4287 and ISO 4288 [67,68]) and instrument restrictions.

The used handheld stylus instrument was unable to capture an entire 40 mm profile in one go. Therefore, multiple line profiles were combined to obtain the required evaluation length. Similarly, due to the sample curvature, multiple shorter areas were captured using the confocal microscopy system and up to five parallel profiles were extracted and combined. This was to enable a smaller measurement z-range to minimise artefacts, as discussed in [69]. Furthermore, from the fringe projection data, parallel lines were combined for the as-built condition. This enabled higher resolution, as data points are reduced when stitching, and even faster measurement speed. The width resulted from the respective instrument field of view (FOV) and is given in Table 3.

Even though the line roughness measurements were not matched exactly as the alignment of profile measurements is difficult, taking an average value for multiple lines within the same marked area and filtering the same bandwidth is assumed to produce comparable results. This procedure is meant to reproduce realistic QA process conditions.

The resolution for stylus measurements depends on multiple factors, therefore, both point cloud density (1.5 μm) and tip size (2.0 μm) are specified in Table 3. The resolution of the confocal microscopy measurements from stitched data was 1.38 μm. Fringe projection achieved an xy-resolution of 3.70 μm Table 3.

#### 2.3.2. Preparation of Measurement Data

Data preparation and evaluation were performed using the MountainsMap^®^ V9 software by DigitalSurf.

The measured areas from random locations (as indicated in Figure 9) were processed as follows. Outliers were removed applying the ’soft’ setting and non-measured points were interpolated. As previously mentioned, parallel profiles extracted from the same areal measurement were stitched to obtain the required evaluation length. This was performed for 7 measured locations per surface condition and measurement method.

#### 2.3.3. Evaluation of Surface Texture Parameters

Data filtering, including form removal, high pass ( $\lambda_{c}$ /μm) and low pass ( $\lambda_{s}$ /μm) filtering, was performed as described in ISO 21920-3 [54]. Applied filtering values per sample are summarised in Table 4. In order to ensure comparability, the same bandwidth was extracted from the data obtained from each method. Rt , the maximum total height, and Ra , the arithmetic mean deviation from the roughness profile, were evaluated on evaluation lengths of 12.5 mm for ChM, 3DS and 3DS+, and 40 mm for the AsB condition.

## 3. Results and Discussion

This section starts out with the comparison and correlation of the results obtained from stylus measurement, fringe projection and confocal microscopy. Subsequently, method-specific challenges are discussed and summarised.

### 3.1. Comparison of Ra and Rt from Confocal, Fringe Projection and Stylus Data

**Figure 10:**
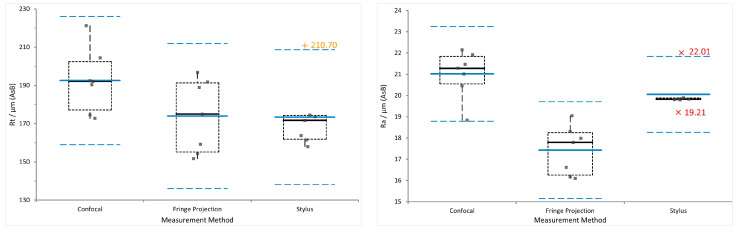
AsB— Rt (**left**) and Ra (**right**) data from confocal, fringe projection and stylus measurements.

**Figure 11:**
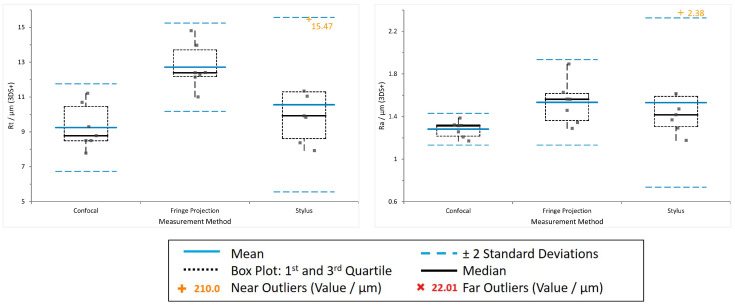
The 3DS+— Rt (**left**) and Ra (**right**) data from confocal, fringe projection and stylus measurements.

Figure 10 and Figure 11 show plots of the data obtained from the three evaluated surface characterisation methods for as-built (AsB)—the ’roughest’—and combined post-processing (3DS+)—the ’smoothest’—surface conditions. Seven data points per sample and method are displayed. In each plot, the blue line indicates the data mean, dashed blue lines mark two standard deviation from the mean (2SD), the dashed box encloses data between the first and third quartile of the data set and the whiskers extending from the box denote highest and lowest values. Outliers are determined with regard to the inter-quartile range (IQR), where near outliers (orange +’s) are defined between 1.5 and 3 IQR and far outliers (red x’s) outside of 3 IQR.

Looking at the data presented in Table 5 and Table 6, especially for Ra , good correspondence across the measurement methods is observed, suggesting that all three methods detect comparable surface quality for each of the three surface conditions (as-built and post-processed).

For the AsB condition, the Ra values from fringe projection are slightly lower. This may be due to apparent profile smoothing (Figure 8) from lower data point density. Additionally, the area covered by fringe projection is the largest, so the variation could also simply be caused by a variation in physical surface properties. Looking at the stylus results, the majority of values shows no variation, indicated by the very narrow box plot. This is caused by measurability issues. Many locations could not be measured due to the tip movement being restricted by surface features. Obtaining seven full profiles on the AsB surface (consisting of 21 individual measurements in total) required more than 30 individual measurements (i.e., almost 1/3 of measurement attempts were unsuccessful). Hence, this very small variation in results is not related to a homogeneous surface quality, but to a pre-selection of measurable locations. A similar effect was observed for the ChM sample, due to the typical crater features (Figure 6). The 3DS and 3DS+ stylus measurements were undisturbed as restricting features were successfully removed during surface treatment.

**Figure 12:**
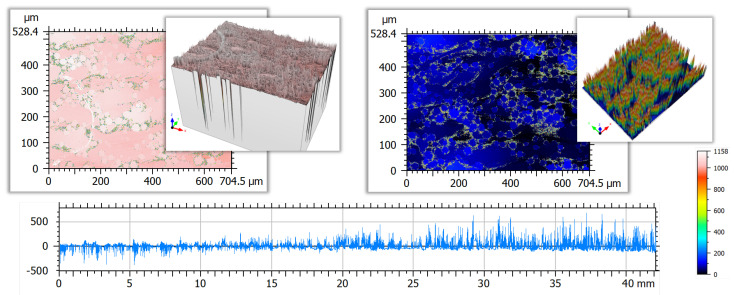
AsB—height distributions of outer left and right images from LSCM measurement showing spike artefacts along particle boundaries (**top**), LSCM profile data after form removal showing spike size variations along the measured profile (**bottom**).

Considering the Rt results, individual Rt values range from 150 μm to 220 μm for AsB, Figure 10. The highest mean value of 193 μm is obtained from the confocal measurements. The parameter itself is designed to give one extreme value, making it highly dependent on the measured location. One possible explanation for the increased values may be that data post-processing did not remove the occurring spike artefacts (see Figure 12) entirely, which has an enormous impact on the extreme value Rt . This matter is addressed in more detail in Section 3.3. Another option is the influence of location and measured area width. The parallel profiles combined to obtain the necessary evaluation length was quite narrow (approx. 500 μm, see Table 3).

**Figure 13:**
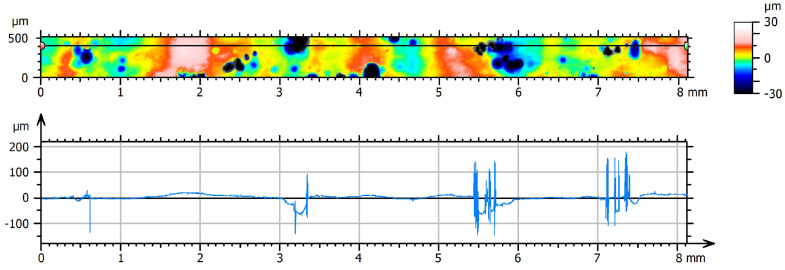
ChM—spike artefacts in ChM raw data (after form removal) from LSCM associated with crater features; location of extracted profile: black line, bordered by red and green markers.

A similar observation is made on the ChM surface, where confocal results have a 44% higher Rt than the fringe projection data. Their 2SD regions still overlap, meaning they are not statistically distinctive applying a 95% confidence interval. Furthermore, the typical crater-like features, as shown in Figure 6, have sharp edges, causing spike artefacts similar to those on the AsB data. These may, again, not be fully removed by data post-processing (see Figure 13). Apparent profile smoothing in fringe projection and stylus measurements as well as reduced valley penetration depth may also play a role in this consideration. For the stylus results, profile flattening by the tip during measurement may contribute to the result, as the contact measurement can have an influence on its result [66].

The variations within one method and from one method to the other illustrate one of the major drawbacks of profile characterisation altogether, namely the lack of reproducibility and surface representation. If areal measurements and characterisation parameters [38] are used instead, a more representative surface portion can be covered and the chance to capture the largest height variation on the examined surface increases. In general, areal surface characterisation is more powerful with respect to the extraction of 3D topography information [16,37], even though limited to line-of-sight data, when using optical measurement methods [17,36,70].

### 3.2. Correlation of Results from Evaluated Methods

Taking a look at the correlation of the data from the evaluated methods across the different surface conditions, good correspondence can be observed, as shown in Table 7. Correlation coefficients of at least 0.984 between the data sets and R^2^-values of more than 0.967 for a linear regression model show that—when filtering the same bandwidth and restricting the measurement area—fringe projection, confocal microscopy and stylus profilometry achieve comparable results despite the scattering of profile parameters. A similar observation was made by Piska et al. [71] in their comparison of stylus and focus variation results for machined surfaces. Independent from the individual method-specific challenges discussed subsequently, this leads to the conclusion that all methods give valid results for the assessed surface conditions and selected parameters. All correlation coefficients (CC) and R^2^-values are given in Table 7.

### 3.3. Discussion of Method-Specific Challenges

In the previous sections, the surface texture characterisation results of four different surface conditions using three measurement methods and various influences on the results were discussed. It was demonstrated that all of the applied measurement systems produced comparable results. In this section, hands-on experiences from measurement and data post-processing are discussed. The qualitative findings with regard to acquisition time, operator effort, surface coverage, reproducibility, operator skill and other aspects are summarised in Table 8 in Section 4.

A common shortcoming of all of the evaluated methods is the lack of re-entrant feature detection capability. Recognition of re-entrant features can be provided by X-ray computed tomography measurements, which are, in addition, able to acquire information on a parts’ bulk quality. Drawbacks are the long acquisition time, limitation of object size and high cost of the system, as well as the requirement of highly specialised operators. However, its potential for a holistic description and understanding of metal AM part quality is undeniable [17,72,73].

This being said, areal measurements do offer a better statistical representation of a surface than 2D profile data and are increasingly applied in academic and industrial contexts. The application of these methods, however, currently requires a higher level of skill than the established stylus method, as not all aspects of the measurements and data post-processing are standardised (yet). Though, the areal surface characterisation standard is continuously being updated and its current version does contain a list of optical methods considered suitable for this purpose.

#### 3.3.1. Laser Scanning Confocal Microscopy (LSCM)

Amongst the assessed methods, confocal microscopy offers the highest resolution (Table 3). A major disadvantage of the method is the measured area in relation to acquisition time. Though this may vary from one confocal instrument to the other, higher resolution measurements generally take longer.

Initially it was intended to capture the entire evaluation length required for a surface condition based on expected roughness in one single measurement. However, looking at the AsB condition, the required evaluation length of 40 mm required a acquisition time of more than 12 h. Additionally, due to the sample’s curvature (Figure 5), a fairly high z-range setting was necessary to capture the height variation across the entire length (the VK9700 system used in this study only allows for one z-range setting for the entire measurement, other instruments may offer the options to apply a variable z-range). In combination with shadowing effects from visible agglomerated powder particles and surface inclination, this resulted in large spike artefacts of up to 0.7 mm in size, as is shown in Figure 12. Cross-section micrographs clearly confirm the spikes being measurement artefacts (Figure 4). Figure 13 visualises that spike artefacts also occurred in the ChM data, caused by the crater features present on the surface.

**Figure 14:**
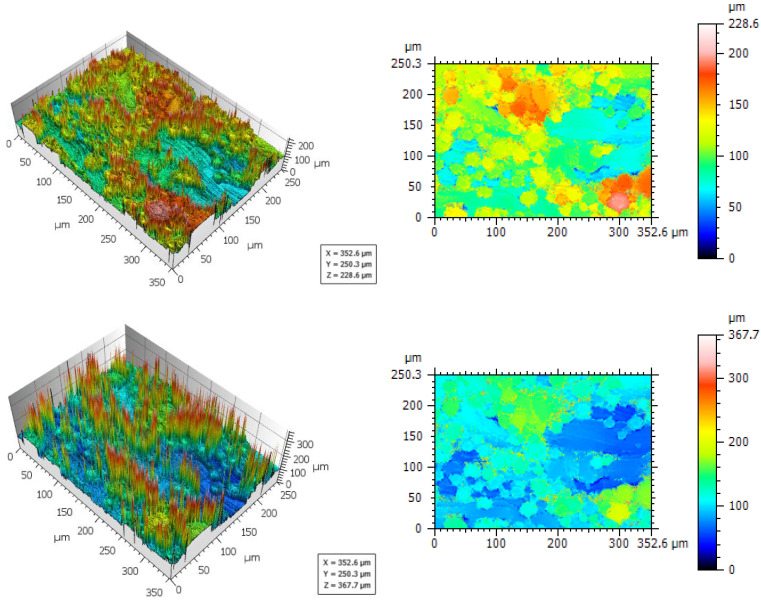
AsB—spike size with variation in preselected measurement z-range from LSCM: Δz = 229 μm (**top**), Δz = 368 μm (**bottom**). Spike size is increased with larger preselected zrange [69].

It is observed from the raw data after form removal that the full-length profile exhibits valley spikes to the left and peak spikes to the right of the measured area (Figure 12). This illustrates that the artefacts depend on the angle of the surface with regard to the light source, meaning, that the distribution of artefacts would likely be different when changing the positioning due to changed surface inclination. The size of the spikes appears to be restricted by the pre-defined z-range specified before the measurement, illustrated by Figure 14, were the same location was measured twice with adjusted z-range setting (part of the figure is reproduced from [69]). The effect of measurement z-range and selected data post-processing setting are discussed in [69].

Attempts to remove those spike artefacts during the data post-processing step resulted in a large number of non-measured points, which is why it was decided to measure shorter areas and combine parallel profiles.

Due to the long duration of measurements it is tempting to capture smaller areas, especially when the aim is to gain a qualitative understanding of the surface texture to be investigated. The issue when capturing smaller areas, however, is shown in Figure 6. Two positions on the ChM samples were measured, showing very different surface features due to its inhomogeneous surface finish. These measurements could be obtained within a relatively short time (15 to 20 min, dependent on measurement z-range). However, they do not succeed to capture a representative portion of the surface to evaluate its quality. When performing areal surface texture measurements, it is essential to select a sufficiently large representative area portion. For profile roughness evaluation, the required length is clearly indicated in the ISO 21920 standard [44,54]. Considering areal characterisation, the ISO 25178 indicates to use at least five times the size of the largest feature of interest [74].

#### 3.3.2. Fringe Projection

Fringe projection is the quickest of the assessed measurement methods (approx. 10 mm × 5 mm in 4 min), but also has a considerably lower resolution than confocal microscopy (refer to Table 3). The achieved lateral resolution of 3.7 µm was also used by Whip et al., when they measured as-built metal AM surface texture [23]. It is comparable to or higher than some resolution values found in the literature for AM surface texture characterisation with focus variation instruments [17,59,75]. Triantaphyllou et al. [59], for example, worked with a lateral resolution of 8 μm on their focus variation instrument. When applying standard profile roughness filter values (Table 4), the resolution reached by the fringe projection instrument is considered sufficiently high for this application.

As for other optical surface topography measurement methods, a drawback of fringe projection is the line-of-sight measurement, meaning re-entrant features (see Figure 4) cannot be detected [36].

Considerable advantages of the method are the quick measurement speed and large area coverage as well as little required data post-processing for this specific application. However, fringe projection instruments do have troubles accurately depicting reflective surfaces, especially when inclined, as found in [76] for machined fatigue specimens. When covering larger areas with the VR3200 system by stitching, the resolution is reduced to decrease the data size. This has to be paid attention to as it can lead to an apparent surface smoothing effect, illustrated in Figure 8.

#### 3.3.3. Stylus Profilometry

When taking stylus measurements, a strong dependence on measured location was observed. Additionally, not every location could be measured due to the tip movement being hindered by particle agglomerations, other surface appearances or due to sample curvature. Measurability is improved when decreasing the movement speed of the tip, as abrupt impact forces when hitting vertical features are decreased. These impact forces may cause damage to the tip itself. Additionally, the measured surfaces may be scratched from the contact measurement [66], possibly compromising mechanical performance or corrosion resistance.

The stylus method is incapable of detecting re-entrant features [17,56,77] and application to complex geometries is restricted by instrument and measurement requirements. The accurate alignment of measurements (direction of individual measurements, parallelity of multiple profiles in same direction) for the applied handheld Mituyoto SJ210 is hardly possible. Due to the maximum available length of 17.5 mm for measurement, rougher surfaces may require manual stitching and data processing with software tools, such as the MountainsMap^®^ software applied in this work. Issues regarding alignment and measurement length may not arise when using fixed frame instruments.

Generally, a stylus profiler captures 2D line profiles, which cannot be considered representative, especially in case of coarse and irregular as-built LPBF surfaces. Furthermore, measurements are hardly reproducible as it is unlikely to match exact locations when performing repeat measurements.

Advantages of this method are, however, that its physical working principle is fully understood and it is entirely standardised (method, instrument and data filtering in ISO 21920-2, ISO 21920-3 and ISO 3274 [44,54,55]). The equipment is affordable and the measurement execution is straight forward, making the method accessible to a broad range of users.

## 4. Conclusions

This work aimed at demonstrating the suitability of three different methods to measure surface topography of as-built and post-processed Ti-64 surfaces from LPBF, and at comparing these methods. The applied methods, confocal microscopy, fringe projection and stylus profilometry, showed good correspondence of results for the characterisation of as-built and wet-chemically post-processed LPBF surfaces, rated by linear correlation coefficients (>0.984) and corresponding R^2^-values (>0.967). For quick reference, a qualitative summary of the method comparison is included in Table 8.

Confocal microscopy offered the highest resolution but was also time consuming. In the AsB and ChM raw data, spike artefacts from feature interaction with the light source (sharp edges, shadowing) occurred, requiring appropriate post-processing.

Fringe projection was the quickest and easiest of the investigated methods regarding measurement and data post-processing. When covering large area portions, reproduction of location when performing repeat measurements is possible. The spatial resolution is lower than for confocal microscopy but is still considered sufficiently high to characterise the investigated surface conditions. The computed Ra values for the AsB condition are lower than for confocal (approx. 20% on mean) and stylus (approx. 15%) methods, which may be attributed to a combination of lower point spacing and physical variations on the sample.

The main disadvantages of the observed stylus method were the data pre-selection due to feature-caused restriction of tip movement and limitation to 2D profile measurement. For the AsB and ChM surface conditions, the measurability was strongly restricted by characteristic features (powder particle agglomerations, craters). For the AsB surface, only two out of three measurement attempts were successful, leading to a significant increase in effort and acquisition time. However, being entirely standardised and handheld instruments being inexpensive, the method is accessible to a large group of users.

To accommodate the use of optical measurement instruments for qualification and QA processes, tolerance values will have to be defined accounting for differences in data acquisition and representation. Exploiting the full scope of advantages of optical systems will ultimately enable a more accurate, precise and reproducible description of surface quality.

Concerning parameters for metal AM surface characterisation, Ra and Rt were used in this study as they are commonly known and accepted in order to demonstrate the methods’ applicability and comparability. Areal parameters, however, do offer information on a larger, more representative surface portion and a statistically more meaningful depiction [16]. The areal equivalents to Ra and Rt according to ISO 28175-2 are Sa and Sz [38]. Within this standard, many other parameters are available. In recent years, areal parameters are increasingly evaluated. However, Todhunter et al. found, that this is especially the case for research institutions and metrology and calibration industry [18].

In order to facilitate industry’s transition from profile to areal characterisation and from stylus to optical instruments, user oriented education and guidance is needed. Therefore, a broader investigation, including methods based on different physical working principles, instruments by different manufacturers and surface conditions from different manufacturing processes, is required for the development of guidelines and standards.

**Table 8:** Summary—comparison of confocal microscopy, fringe projection and stylus profilometry for metal AM surface texture characterisation.

	Confocal Microscopy	Fringe Projection	Stylus Profilometry
*Acquisition time*	very long–large z-range required to capture entire evaluation length in one meas.	short–larger FOV, stitching of few images for full evaluation length	long (multiple individual line measurements necessary, restricted tip movement due to surface features)
*Lateral/spatial resolution*	high	sufficient	sufficient
*Representative surface coverage*	yes	yes	no
*Linear/areal parameters*	both	both	linear
*Standardization*	listed as suitable method	listed as suitable method	fully standardized (instrument, data processing, parameters)
*Physical principle*	optical/non-contact; layering of in-focus z-data	optical/non-contact; pattern projection, triangulation	contact measurement
*Surface damage*	no	no	possible
*Detection of re-entrant features*	no	no	no
*Reproducibility*	medium/high—localisation of small area portions is possible but challenging using macroscopic markers	high—large area portions can been measured and located by means of macroscopic markers	low—individual lines unlikely to be located when repeating measurement, surface may be influenced by first (contact) measurement
*Measurability*	good	good	restriction of tip movement (powder particle agglomerations, craters), limited z-range (handheld devices)
*Operator skill*	high level of proficiency required to select measurement settings appropriately and perform data processing	medium high level of proficiency required to select measurement settings appropriately and perform data processing	handheld devices are easy to use, process is fully standardised, alignment of multiple (parallel) measurements is highly difficult
*Operator effort*	medium/low – complex initial setup, automated measurement	low – fairly straightforward initial setup, automated measurement	labour-intensive – every location has to be selected and measured individually (for handheld devices)

## Figures and Tables

**Table 1:** Samples with corresponding surface conditions.

Sample	Size (Approx.)	Surface Treatment	Treatment Duration
AsB	42 mm × 25 mm	n/a	n/a
ChM	25 mm × 40 mm	Chemical milling	20 min
3DS	30 mm × 40 mm	3D SurFin^®^	15 min
3DS+	30 mm × 45 mm	3D SurFin^®^ +	15 min + 20 min
		Chemical milling	

**Table 2:** Applied surface treatments [8].

Treatment Process	3D SurFin	Chemical Milling
Temperature (range)/°C	80	55
Removal rate (range)/μm/min	8	12
Bath size/L	100	17
Bath components	$NH_{4}F$ , $H_{2}SO_{4}$ , $C_{7}H_{8}NNa_{3}O_{6}$	HF , $HNO_{3}$
	water based	water based

**Table 3:** Measurement details for each method and sample.

Method		Evaluation Length/mm	Measured Area Length/mm	Measured Area Width/mm (Approx.)	Magnification	Lateral Resolution/μm *for Stylus: Point Distance | Tip Diameter*	Approx. Acquisition Time/min
**Confocal**	AsB	40.00	7 × 9.36	0.50	20x	1.38	380
Keyence VK9700	ChM	12.50	7 × 8.12	0.50	20x	1.38	80
	3DS	12.50	7 × 15.54	0.50	20x	1.38	60
	3DS+	12.50	7 × 17.39	0.50	20x	1.38	40
**Fringe projection**	AsB	40.00	7 × 10.22	5.17	80x	3.70	12
Keyence VR3200	ChM	12.50	7 × 3.61	2.84	80x	3.70	4
	3DS	12.50	7 × 3.61	2.84	80x	3.70	4
	3DS+	12.50	7 × 3.61	2.84	80x	3.70	4
**Stylus**	AsB	40.00	21 × 17.50	n/a	n/a	1.50 | 2.00	70
Mitutoyo SJ-210	ChM	12.50	7 × 15.00	n/a	n/a	1.50 | 2.00	25
	3DS	12.50	7 × 15.00	n/a	n/a	1.50 | 2.00	15
	3DS+	12.50	7 × 15.00	n/a	n/a	1.50 | 2.00	15

**Table 4:** Filters applied to measurement data of all methods for each sample [54]: high pass filter $\lambda_{c}/$ mm and low pass filter $\lambda_{s}/\mu$ m.

Sample	$\lambda_{c}/$ mm	$\lambda_{s}$ /μm
AsB	8.0	25.0
ChM	2.5	8.0
3DS	2.5	8.0
3DS+	2.5	8.0

**Table 5:** Summary of results— Rt .

Rt		AsB	ChM	3DS	3DS+
*Confocal*	Mean	192.53	71.27	29.52	9.25
N = 7	St.-dev.	15.52	10.47	6.87	1.16
	% St.-dev.	8.06%	14.69%	23.28%	12.57%
*Fringe projection*	Mean	173.97	48.66	34.23	12.71
N = 7	St.-dev.	17.57	13.69	5.08	1.17
	% St.-dev.	10.10%	28.12%	14.85%	9.20%
*Stylus*	Mean	173.40	41.75	27.22	10.56
N = 7	St.-dev.	16.33	9.42	2.28	2.32
	% St.-dev.	9.42%	22.57%	8.37%	21.94%

**Table 6:** Summary of results— Ra .

Ra		AsB	ChM	3DS	3DS+
*Confocal*	Mean	21.02	5.78	4.11	1.28
N = 7	St.-dev.	1.03	0.85	0.66	0.00
	% St.-dev.	4.90%	14.68%	15.98%	0.00%
*Fringe projection*	Mean	17.43	5.10	5.04	1.53
N = 7	St.-dev.	1.05	1.09	0.73	0.19
	% St.-dev.	6.05%	21.30%	14.55%	12.11%
*Stylus*	Mean	20.06	4.65	3.91	1.53
N = 7	St.-dev.	0.83	0.48	0.38	0.37
	% St.-dev.	4.12%	10.29%	9.75%	24.03%

**Table 7:** Correlation coefficients (*CC*) and *R*^2^ of stylus profilometry, confocal microscopy and fringe projection.

		Confocal		Fringe Projection		Stylus	
		*CC*	*R* *^*2*^*	*CC*	*R* *^*2*^*	*CC*	*R* *^*2*^*
**Confocal**	*CC*			0.987		0.992	
	*R^2^*				0.969		0.984
**Fringe**	*CC*	0.987				0.984	
**Projection**	*R^2^*		0.969				0.967
**Stylus**	*CC*	0.992		0.984			
	*R^2^*		0.984		0.967		

## Data Availability

Data are available from the corresponding author upon request.
